# The PRECICE magnetic IM compression nail for long bone nonunions: a preliminary report

**DOI:** 10.1007/s00402-019-03225-4

**Published:** 2019-06-19

**Authors:** Austin T. Fragomen, David Wellman, S. Robert Rozbruch

**Affiliations:** 1 Weill Medical College of Cornell University, New York, USA; 2 Limb Lengthening and Complex Reconstruction Service, Hospital for Special Surgery, New York, USA; 3 Orthopaedic Trauma Service, Hospital for Special Surgery, 535 East 70th Street, New York, NY 10021 USA

**Keywords:** Nonunion, Compression nail, Magnetic, PRECICE, Lengthening nail

## Abstract

**Introduction:**

The magnetic intramedullary (IM) compression nail is capable of providing sustained compression for the treatment of nonunions of long bones. This ability was previously only possible with the use of external fixation. We asked the following questions: How effective is the IM compression nail at achieving union? How do we know when adequate compression has been attained? Which types of nonunions are good candidates for this treatment?

**Materials and methods:**

Fourteen patients with nonunions of the tibia (5) or femur (9) were treated with the PRECICE IM compression nail. The average age was 49 years number of previous surgeries was 1.9, 7 were atrophic and 7 normotrophic, 3 were metaphyseal and 11 diaphyseal. All PRECICE IM nails were pre-distracted prior to implantation. Compression was applied post-operatively until the locking bolts were seen on X-ray to be bending or the nail was no longer shortening despite applying the external magnet.

**Results:**

Union was achieved in 13/14 cases. The time to union was 24.5 weeks (range 11–60). The two proximal tibia metaphyseal nonunions, both deformed into varus (4°) and flexion (10°) after compression was applied with one failing to unite. The distal tibia metaphyseal and diaphyseal nonunions did not deform upon compression. Three patients had positive cultures and were treated with IV antibiotics for 6 weeks followed by 3 months of oral suppression without subsequent infection. No mechanical nail failures were seen.

**Conclusions:**

The IM compression nail was successful at applying compression, preventing deformity, and obtaining union in all diaphyseal and in distal tibia metaphyseal nonunions. Signs of active compression are bending of the locking bolts and failure of the nail to shorten. Proximal tibia metaphyseal nonunion may not be suited for this treatment.

## Introduction

Nonunions of the femur and tibia occur in 2.5–10% of fracture cases [1, 2] and can be difficult to manage. Nonunion treatment often requires several surgeries, creates lost work time, and results in both financial burden [3, 4] and a poor health-related quality of life for the patient [5, 6]. The etiology of fracture nonunion is vast and multifactorial [7]. Treatments have primarily been surgical. Open bone grafting with plating has yielded high rates of union [8, 9], but plating is an extensive surgical intervention. Exchange intramedullary (IM) nailing has been the mainstay of treatment for diaphyseal nonunion in the femur and tibia with success rates ranging from 53 to 100% [10, 11] and 72 to 92% [12], respectively. Part of the range in union outcomes may be due to poor bone contact at the nonunion docking site [12]. The value of compression in the biomechanics of osteogenesis has been long recognized [13, 14]. IM tibial and femoral nails allow for mild compression to be delivered at the time of surgery [15, 16] but provide no mechanism to sustain the compression over time. Circular external fixation provides an alternative method for nonunion management with the advantage of providing sustained compression during the recovery period but requires wearing the frame for a prolonged post-operative course [17–19]. Surgeons have gone to great lengths to achieve compression, even integrating IM nails with circular fixators as in the compression over a nail technique [20]. The advent of the PRECICE (NuVasive Specialized Orthopedics, San Diego, CA, USA) magnetic IM compression and distraction nail has created a treatment option that combines the convenience of IM nailing with the sustained compression that is ideal for fracture healing. Watson et al. [21] published a series of at-risk humeral fractures treated with the magnetic intramedullary compression nail (MICN) that demonstrated 100% union. A case report of a patient who had failed circular fixation-assisted compression for a tibial nonunion demonstrated rapid healing when treated with the MICN [22]. The present study asked the following questions: (1) How effective is the MICN at achieving union? (2) How do we know when adequate compression has been attained? (3) Which types of nonunions are good candidates for this treatment?

## Materials and methods

### Study group

Fourteen patients with nonunions of the tibia (5) or femur (9) were treated with the PRECICE MICN. Patient demographics are listed (Table 1). Eleven of the 14 patients had post-traumatic fracture nonunion of either the femur or tibia, 1 patient had nonunion of a femoral allograft used for reconstruction after tumor resection, and 2 had femur osteotomy nonunions. Nonunion was confirmed on CT scan when questionable. Ten of 14 patients had failed prior attempts at nonunion repair (average 2.3 previous surgeries, range 2–5). Indications for MICN implant used in this practice included cases of nonunion in long bones where treatment with standard internal fixation or circular external fixation was already attempted or such treatment could be expected to fail. These selective indications are responsible for the limited size of this cohort. All MICNs were pre-distracted an average of 13.5 mm (range 10–18) prior to implantation. Five of the patients had been treated for fracture-related infection and were thought to be infection free at the time of MICN insertion. All patients received a standard pre-operative workup including serum ESR, CRP, and WBC counts. Active infection was a relative contraindication for this surgery. Need for hardware removal, deformity correction, and for later limb lengthening due to limb length inequality were assessed. Appropriateness of treatment was considered. Access to and experience with state-of-the-art hexapod external fixation and internal fixation from multiple vendors were unlimited, and surgeons chose to use the MICN strictly based on clinical experience.

**Table 1:** Patient demographics

Case #	Age	Sex	Seg	Etiology	Comorbidity	Fx type (GA)	Rx (#; type)	Time: initial Rx failure to ICN (months)	NU type	AIM
1	49	M	TB	Trauma	Smoking, opioid, treated infection	O, 3A	5; IMN, CP, BT	72	N	11
2	27	M	TB	Trauma	Highly contaminated Fx	O, 3A	2; CP, CEF	14	A	11
3	54	M	FR	Tumor resection	treated infection	C, Fx Allogrft	2; IMN and CP	84	N	5
4	27	M	FR	Trauma	Obesity, treated infection, bone defect	C	2; IMN	8	A	8
5	35	M	FR	Trauma	Smoking	O, 3A	1; IMN	23	N	5
6	71	M	TB	Trauma	DM2, ESRD, CHF, treated infection, bone defect	O, 3B	2; IMN, CEF	15	A	19
7	47	F	FR	Trauma	DM1, opioid, blindness, osteoporosis	C	1; IMN	15	N	7
8	40	F	FR	Osteotomy	Rickets	C	1; IMN	10	A	6
9	83	F	FR	Trauma	Treated infection, DM	C	2; IMN and MEF	60	A	8
10	24	M	FR	Trauma	none	C	2; IMN	13	A	5
11	56	F	FR	Osteotomy	none	C	2; IMN	13	N	5
12	75	F	FR	Trauma	Smoking, opioid	C	2; CP and IMN	16	A	6
13	41	M	TB	Trauma	none	0, 3A	1; IMN	10	N	4
14	62	M	TB	Trauma	RA, enbrel, opioid	C	2; CP, BMAC	13	N	8
Mean	49.3	M- 9/14	FR-9 TB-5	-	-	C-9 O-5	1.9	26.1	A-7/14	7.7

*Case #* patient case #, *M* male, *F* female, *Seg* segment, *T* tibia, *F* femur, *Opioid* opioid addiction/chronic pain, *DM1* diabetes mellitus type 1, *DM2* diabetes mellitus type 2, *ESRD* end stage renal disease, *CHF* congestive heart failure, *Fx* fracture, *GA* Gustilo–Anderson [23], *O* open fracture, *C* closed, *Rx* treatment, *IMN* intramedullary nail, *CP* compression plating, *BT* bone transport, *CEF* circular external fixation, *ICN* internal compression nailing, *NU* nonunion, *N* normotrophic, *A* atrophic, *AIM* AIM score [24] with 7.7 representing moderate complexity

### Surgical technique

Existing hardware was removed either completely or at minimum from the path of the planned IM nail. In most cases, the nonunion site was approached with an open technique, debrided to bleeding bone, and irrigated. Cultures and pathology specimens were obtained. A two-pin external fixator was applied posterior to the path of the nail to control rotation and the reduction. Blocking screws were inserted to prevent deformity in all metaphyseal nonunions and in some diaphyseal cases. These screws were inserted prior to reaming to help direct the reamer. The nail entry point was localized with a Steinman pin, and a 12-mm cannulated acorn reamer was used to start the path of the nail. A rigid hand reamer was needed in many cases to recreate the IM canal across the sclerotic nonunion site prior to ball-tipped guide wire insertion. Once the ball-tipped wire was in position, reaming was performed using flexible reamers. Overreaming by 2 mm was done to ensure the bone’s ability to slide over the nail and compress across the nonunion site. The guide wire was removed, and the non-cannulated nail was inserted gently. Rough impaction or excessive bending of the nail can damage the internal gears and render the nail ineffective. If the bone was too tight to insert the nail, then further reaming was performed. Locking of the nail was performed in a standard fashion with a combination of a jig for the screws near the entry and free-hand technique for the screws at the far end.

**Fig. 1:**
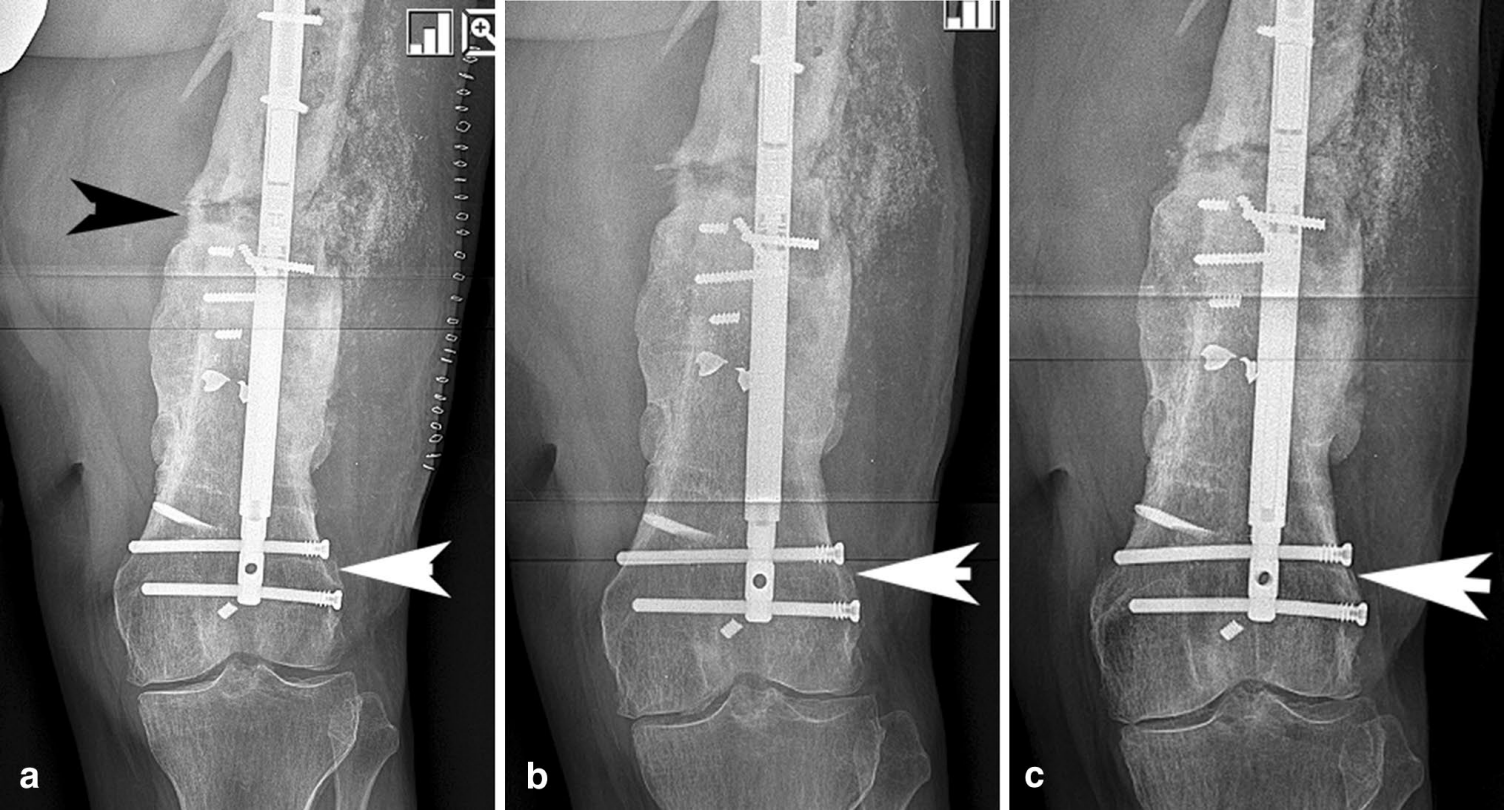
**a** This AP X-ray shows a patient (case #3) with bending of the distal locking bolts (white arrow) indicating strong compression at the nonunion site (black arrow). **b** The same patient underwent radiographic examination one month later with visible loss of bending of the screws (arrow) indicating a loss of compressive load at the bone ends. Additional compression of 2 mm per day for 2 days was applied after this visit. **c** The same patient had X-rays taken one month later demonstrating screw bending (arrow), evidence that compression was present at the nonunion site

Using the C-arm fluoroscopy, the internal magnet was localized and marked on the skin. A loose stitch was placed through the skin at this point as a more permanent indicator of where to place the external remote control (ERC) magnet post-operatively. The ERC placed on the thigh or leg spins the magnet in the nail leading to gradual compression. Compression was applied in the operating room in two cases and post-operatively in all cases until the locking bolts were seen to be bending under the force of the nail or the nail was no longer shortening despite applying the ERC (Table 2). The telltale sign that compression was present was bolt deflection (bending) as seen on radiographs. If bolt bending was not seen on a post-operative radiograph, then more compression was applied. (Fig. 1a–c) Since this system was originally designed for bone lengthening, to compress the nail the magnet was programed to lengthen in the opposite direction. For example, for an antegrade femur nail to compress, the ERC was programed for a retrograde lengthening. Compression intervals were set for 1 mm per session.

**Table 2:** Compression schedules used

Case #	Intra-op compression	POD 2	POD 3	POD 4	Home	Office	Total compression
1	0	2	2	1	0	1 PRN	8
2	2	2	2	0	0	1 PRN	8
3	0	0	0	0	2/day		10
4	0	1	1	1	0	1 PRN	10
5	0	0	0	0	3		3
6	0	2	2	2	2/day		10
7	0	2	2	2	1		7
8	0	2	2	1	2/day		11
9	0	1	1	1	0	1 PRN	9
10	0	1	1	1	0	1 PRN	6
11	1	1	1	1	1/day		13
12	0	2	2	2	0	2 PRN	10
13	0	2	2	2	0	2 PRN	10
14	0	2	2	2	3	1 PRN	18
Mean	0.21	1.4	1.4	1.1			9.5

The applied compression in this table refers to the quantity and frequency of ERC applications. It is a measure of how much compression the patient attempted to deliver to the nail and bone

Patients were placed on venous thromboembolic (VTE) prophylaxis, typically rivaroxaban 10 mg, starting post-operative day 2 and lasting for 2 weeks. Weight bearing was allowed but varied by patient bone quality, surgeon experience, and surgeon preference (Table 3). Patients were followed monthly until consolidation. An ERC was available in the outpatient clinic, and additional compression was applied as indicated at these follow-up visits.

**Table 3:** Peri-operative details results

Case #	Nail diamtr (mm)	Nail Pre-distraction (mm)	WB (% BW)	Cmprs applied (mm)	Distancenail shortened (mm)	Distance bone shortened (mm)	Bolt bending (deg)	FRI +	Time to union (wks)	Final LLD (mm)
1	12.5	10	50	8	8	5	2		20	25
2	10.7	10	50	8	2	0	1		12	19
3	12.5	15	50	10	10	7	6		45	112
4	12.5	15	50	10	10	3	4	Y	32	100
5	12.5	15	AT	3	3	2	0		19	0
6	12.5	15	AT	10	9	5	5		15	60
7	12.5	13	50	7	4	2	3		11	25
8	10.7	13	AT	11	6	4	3		34	51
9	12.5	13	50	9	8	4	0		60	50
10	12.5	13	70lbs	6	6	3	6		13	10
11	12.5	13	70lbs	13	9	3	5		13	20
12	12.5	13	AT	10	6	0	0		28	40
13	10.7	13	AT	10	4	0	0	Y	16	0
14	12.5	18	70lbs	18	10	5	0		–	19
Mean	–	13.5	–	9.5	6.7	3.1	2.5	–	24.5	37.9

*Diamtr* diameter, *Cmprs* compression, *WB* weight bearing, *%BW* % of body weight allowed, *AT* as tolerated, *wks* weeks, *XR* X-ray, *FRI* fracture-related infection, *LLD* limb length discrepancy

### Study design

**Fig. 2:**
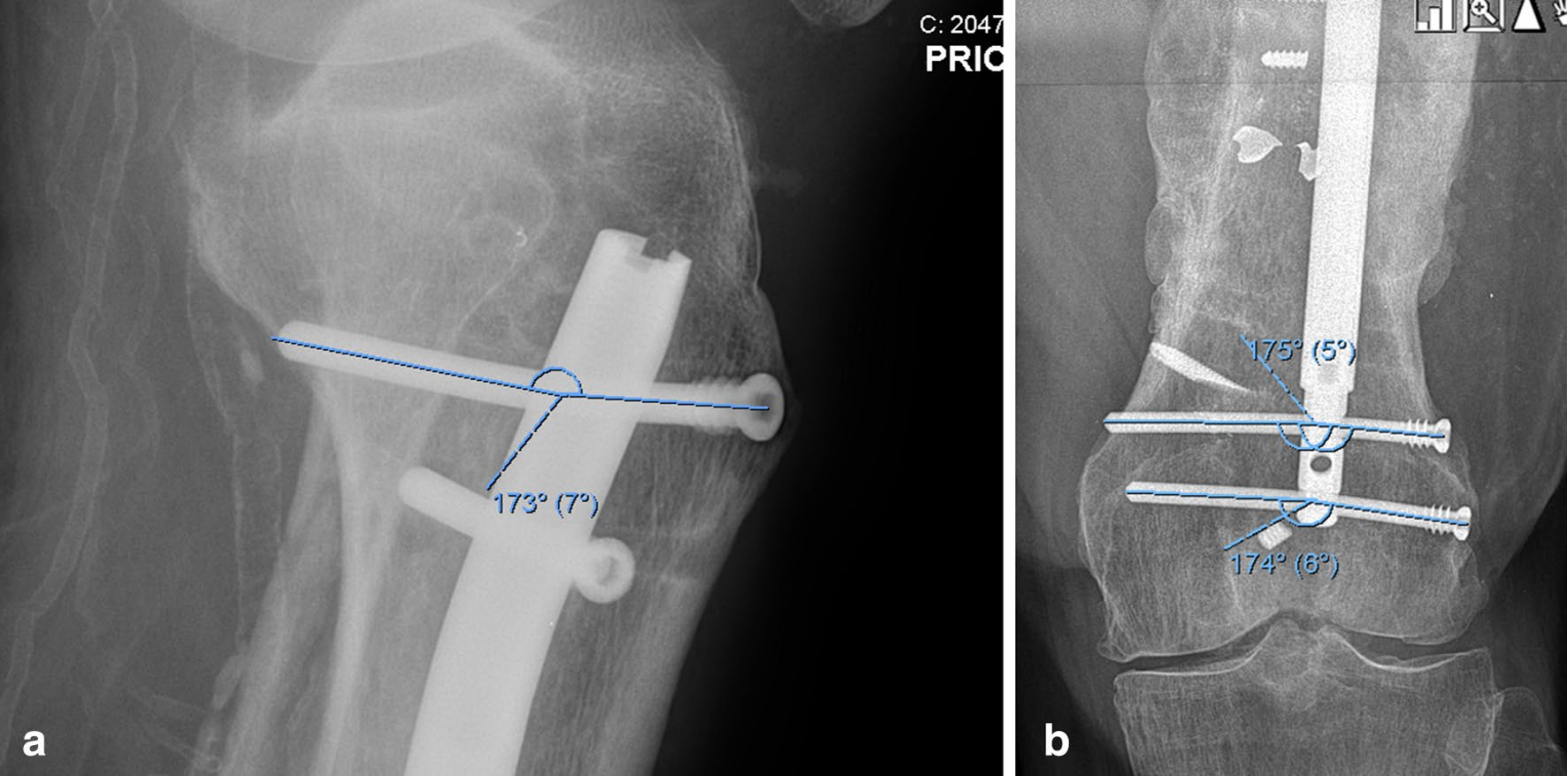
**a** The amount of screw bending can be quantified using an angular measurement. In this case the proximal locking bolt is bending 7°. **b** In this radiograph the bolts are bending 5° and 6°

A retrospective analysis was performed on a consecutive series of 14 patients treated by 3 surgeons at 1 academic orthopedic center in an urban setting. Inclusion criteria for this study included treatment of an established nonunion of a long bone with an MICN. Patients that had the MICN used for purposes other than nonunion repair were excluded. The surgical details were recorded (Table 3). Outcome measures included successful bony union with the implant and time to union. Union was defined as bridging callus on three of four cortices on radiographs or on CT scan. The amounts of compression applied, distance the nail shortened, distance the bone shortened, and angle of locking bolt bending were measured. (Fig. 2a, b) All complications were recorded. AIM scores were collected to gauge the complexity of the impending reconstruction [24].

### Statistics

Statistical analysis was not employed for this limited study. Any conclusions for significance would be unreliable in this cohort.

## Results

Union, as defined by bridging callus on 3 of 4 cortices on either radiographs or CT scan, was achieved in 13/14 cases. The time to union was 24.5 weeks (range 11–60). Follow-up was carried to final union or revision surgery in all cases. The average follow-up was 19 months (range 6–33). Two patients had less than 12-month follow-up. One united after 4 months. The other was censured at 8 months due to persistent nonunion, increasing progressive deformity, and loose hardware that required revision to external fixation.

**Fig. 3:**
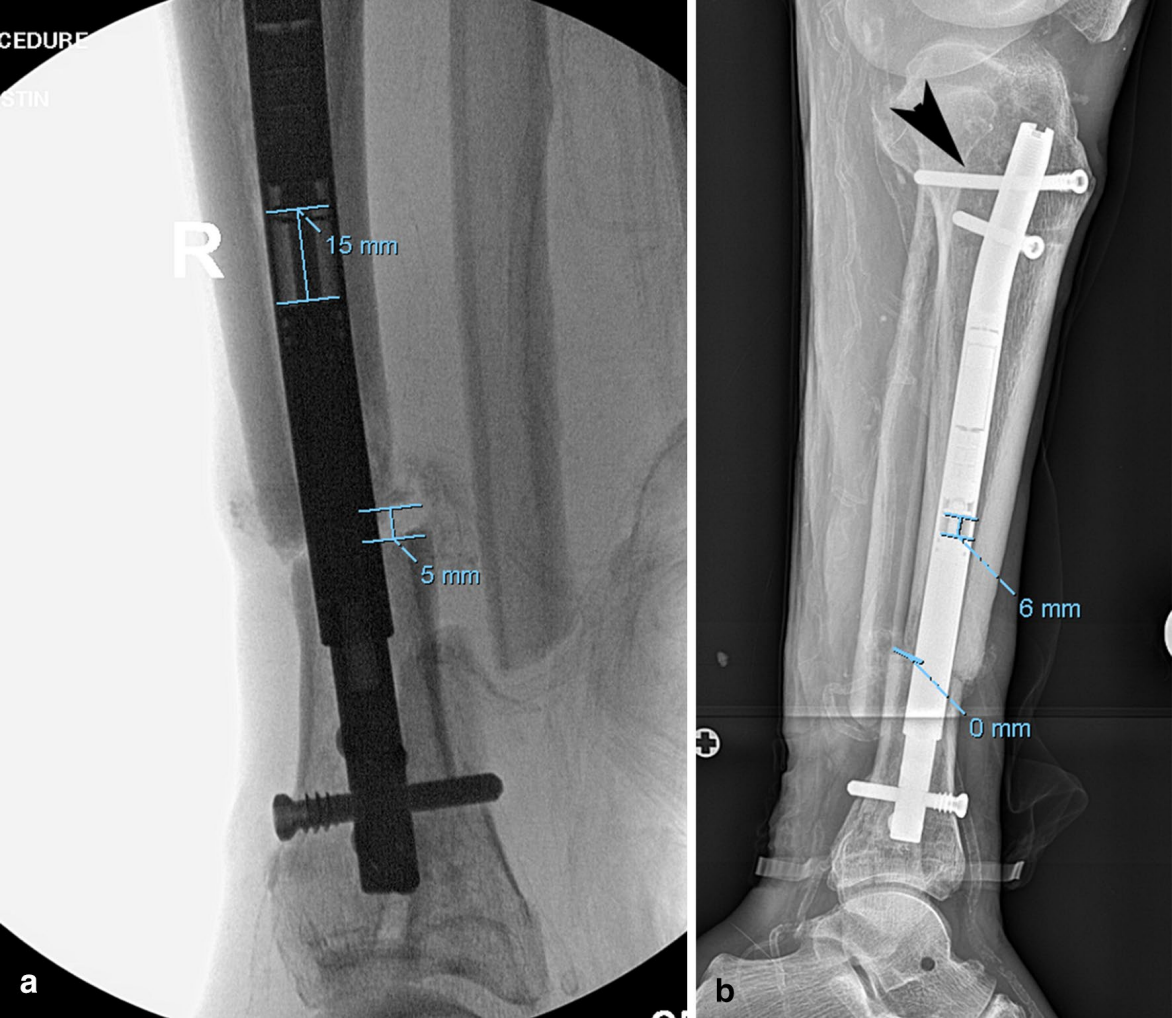
**a** This radiograph is from case #6. The nail is predistracted 15 mm, and there is a bone gap of 5 mm at the nonunion site. **b** The nail has shortened by 9 mm with a residual 6 mm of potential space for additional compression. There is no space at the nonunion site. The proximal bolt (black arrow) is bending

On average, the ERC was used to compress the nail 9.5 mm (range), the nail actually shortened 6.7 mm (range), the bone shortened 3.1 mm (range), and the locking bolts deflected 2.5° (Table 3, Fig. 3a, b). Limb length discrepancy (LLD) after nonunion treatment averaged 3.7 cm (range 0–11.2 cm).

The two proximal tibial metaphyseal nonunions both deformed into varus and flexion after compression was applied with one failing to unite (Table 4). This deformity occurred despite the appropriate use of blocking screws [25]. The patient who failed to unite also had backing out of the locking screws and was revised with a hexapod frame. The distal tibia metaphyseal and the diaphyseal nonunions did not deform upon compression. Two patients (Cases #4 and 13) had positive intra-operative cultures and were treated with IV antibiotics for 6 weeks followed by 3 months of oral suppression without subsequent infection. One patient (Case #2) had an early post-operative infection at the nonunion incision site. He was treated with irrigation and debridement, IM nail retention, 6 weeks IV antibiotics, and oral suppression until union after which point all antibiotics were terminated. No mechanical nail failures were seen through a range of weight bearing loads with 11/14 patients bearing 50% body weight or greater immediately after surgery (Table 5).

**Table 4:** Diaphyseal vs. metaphyseal NU

Patient	Location	Distance from closest joint (mm)	Deformity after compression	Union
1	M	94 Knee	MPTA decreased 4°, PPTA decreased 3°	Y
2	D	270	None	Y
3	D	184	None	Y
4	D	172	None	Y
5	D	215	None	Y
6	M	74 Ankle	None	Y
7	D	153	None	Y
8	D	122	None	Y
9	D	209	None	Y
10	D	182	None	Y
11	D	290	None	Y
12	D	154	None	Y
13	D	233	None	Y
14	M	91 Knee	MPTA decreased 4°, PPTA decreased 18°	N
Mean		174.5		13/14

*NU* nonunion, *M* metaphyseal, *D* diaphyseal, *MPTA* medial proximal tibia angle, *PPTA* posterior proximal tibial angle

**Table 5:** Complications

Complication	*N* (Case #)	Management and outcome
Positive intra-op Cx	2 (Case #4 and 13)	Nail retained, 6 weeks IV ABX, oral suppression until union, no recurrence after union
Post-op infection	1 (Case #2)	Occurred 2 weeks post-op: nail retained, I&D performed, 6 weeks IV ABX, oral suppression until union, no recurrence after union
DVT	0	
Implant failure	0	
Nonunion	1 (Case #14)	Revised with hexapod circular external fixation
Compression-induced deformity	2 (Cases #1 and 14)	Case #1 had no intervention and resulted in malunion. Case #14 was revised and realigned

*ABX* antibiotics, *I&D* irrigation and debridement

## Discussion

The MICN offers a novel, all-internal method to induce healing of recalcitrant long bone nonunions through sustained compression. The optimal indications for this technology will be culled over time for maximum impact and cost optimization. This study does not prove that compression nailing is superior to simple exchange nailing or that it prevents further surgery. If found to be more effective than simple exchange nailing with a classic static nail for complex nonunions in a future controlled trial, the MICN implant’s expense may be offset by the savings of avoiding additional surgery. A recent investigation showed that the same implant used for lengthening introduced no additional cost in the medical system when compared with lengthening over nail which required an additional ambulatory surgery [26]. When compared to a prolonged course wearing a hexapod circular fixator which averages 216 days [18], the MICN with WBAT ambulation may result in earlier return to work, and the cost of the two options may be equivalent; however, there is no data to date to confirm this. The risk of refracture may be less with a IM nail than after immediate removal of an external fixator.

The primary outcome measure was the MICN’s ability to unite nonunions of the femur and tibia. Thirteen of 14 patients united with this treatment over an average of 24.5 weeks. This cohort included many recalcitrant nonunions for which rapid consolidation was not expected. Patients were allowed to weight bear on the implant, and many returned to work by 6 weeks after surgery. Unlike circular fixation reconstructions, the time spent waiting for union was not as cumbersome for the patient. We expected to find a correlation between degrees of screw bending and time to union, but this study lacked the power for this analysis.

The mechanics of applying compression was studied in this group of patients. The goal was simply to compress the nonunion site until bending of the locking screws was seen on radiographs. While the average amount of compression applied to the nail with the ERC was 9.5 mm, the distance that the nail actually shortened was only 6.7 mm. This difference can be explained by the increasing resistance to shortening at the bone docking site resulting in a counter force that the gears in the nail were unable to overcome. Once the bone was well compressed, the external magnet could no longer activate the nail. There was no evidence that any additional attempts to compress the nail beyond this point damaged the implant in any way. In fact, in one patient (Case #4) the same nail was used for a subsequent lengthening without mechanical problems by creating an osteotomy around the nail after the nonunion was healed. The average distance the bone shortened at the nonunion site was 3.1 mm. With the nail shortening 6.7 mm and the bone moving only 3.1 mm, the difference represents the amount of screw bending which allowed the nail to continue shortening when the bone ends were already opposed. The average angle of screw bending was 2.5°. The bending of the screws is the only objective indication of the presence of compression at the bony interface. The degree to which a screw will bend is highly variable and is a function of not only the force applied to it but also: the length of the screw, whether the nail rests in the center of the screw or at one end of the screw, the diameter of the screw (5.0 mm vs. 4.0 mm), etc. A subjective sign of bone compression is pain. The patient will typically experience pain at the nonunion site during and after a compression adjustment with the external magnet. This can last from minutes to days. In one patient, the screws were not seen to bend (Case #13), yet additional ERC compression was painful, and the nail failed to shorten. The failure of the nail to shorten after applying ERC compression is due to strong resistance at the nonunion site, and is therefore another metric to confirm the presence of compression.

Based on this preliminary study, the types of nonunions that may be best suited for the MICN technique are femoral and tibia diaphyseal and distal tibial metaphyseal. Proximal and distal femoral metaphyseal nonunions were not evaluated, and we cannot comment on the use of this technique for these locations. Proximal tibia metaphyseal fractures are difficult to treat with IM nailing, and it follows that nonunions in this location are equally challenging. The compression nail is unique in that it exacerbates the tendency for the proximal fragment to bend medially leading to varus and flexion as the nail pulls the fragments together. The use of blocking screws posterior to the nail and medial to the nail near the nonunion site did not prevent this. Radiographic comparison taken pre- and post-compression showed migration of the nail at the entry point in the proximal tibia suggesting a possible explanation. Osteopenia would likely intensify this nail drift phenomena. The types of nonunions that will benefit from this method need further study.

The complications included one deep infection (Case #2) 2 weeks after conversion from a hexapod frame to the ICN despite a 1-month “frame holiday”. This was treated with open I&D, culture-specific antibiotics (6 weeks IV and 6 months PO), and nail retention. Two other patients were found to have positive intra-operative cultures at the time of nonunion bone debridement and nail insertion. These fracture-related infections [27] were treated with 6 weeks of culture-specific antibiotics, and then oral suppression until bony union. Amorosa et al. found that benign-appearing nonunions had a 28% chance of yielding positive cultures; fortunately, few of those required implant removal [28]. None of our patients required implant removal to control infection.

Limb length discrepancy (LLD) is an expected outcome of any compression technique. (Table 3) Three patients in this series have had subsequent limb lengthening surgery. In one case (Case #4), a corticotomy was made around the nail and the same implant was used. The regenerate was notably quite slow to form (BHI 2.1mo/cm). The patient who had the post-operative infection (Case #2) underwent osteoplasty lengthening 1 year later with a new IM lengthening nail. Although the implanted compression nail could have been used, it was removed due to concerns about chronic contamination from the previous deep infection. A third patient (Case #3) underwent lengthening osteoplasty in the distal femoral metaphysis which required removal of the antegrade compression nail and insertion of a retrograde lengthening nail. Most of the patients with LLD were too metabolically challenged, and therefore poor candidates, for lengthening surgery (Cases #1, 4, 6, 7, 8, 9, 12, and 14). They were treated with shoe lifts. Other healthy patients were simply elected to use a shoe lift (Cases #10 and 11). An alternative approach to nonunion repair and LLD that entails creation of an osteotomy adjacent to the nonunion site to redistribute stress and heal the nonunion was attempted in 1 patient. (Case #5) He underwent osteotomy adjacent to a stiff nonunion in the femur to induce healing at the nonunion site as well as to lengthen the femur through the new osteotomy site. The nail lengthening mode was used to reestablish limb length through the osteotomy. Although the adjacent nonunion was stiff enough that it did not distract during lengthening, unfortunately, it failed to unite. Once the lengthening regenerate bone was mature at the lengthening site, the nail was compressed to facilitate healing at the nonunion site resulting in a strong union.

Bone defects are a common sequela of nonunion surgery. If these defects can be acutely shortened (Cases #4, #9), then the MICN can be used to compress the repair site. The amount of shortening that can be done acutely depends on the soft tissue compliance and neurovascular function. At the time of this study, pre-distraction of the nail was limited to 15 mm so it could not gradually shorten a defect greater than a few millimeters. Recently, a “rapid distraction” tool has become available allowing for 8 cm of pre-distraction which can handle a gradual shortening of several centimeters followed by compression upon docking. How much shortening a patient can tolerate is patient specific. Young and active adult patients will use a shoe lift for LLD of less than 1 cm and will often want lengthening surgery for greater limb length inequality. This technique can be used in either scenario since a staged lengthening surgery can be performed in the same bone. Patients who are poor lengthening candidates can be treated with a shoe lift for any LLD.

Large bone defects may require bone transport rather than shortening which can now be accomplished with an all-internal approach using the novel bone transport nail (PRECICE, NuVasive Specialized Orthopedics, San Diego, CA, USA) or the plate-assisted bone transport technique [29]. Both of these transport methods rely on compression at the docking site, and the preliminary findings of the present study will improve the understanding of the mechanics of MICN compression universally.

The retrospective nature of this single cohort study is a limitation. We were unable to rigorously study the clinical judgement indices used to decide which patients are treated with this MICN technique and which were managed with a different approach. A randomized controlled trial is needed to prove any of the observations noted in this study. The trail would need to compare MICN to simple exchange nailing to show the actual value of sustained compression. Anecdotally, some of the patients studied (Cases #3, #4) lost compression early in treatment and had no progression of union over 3 months. Compression was added and the bones united quickly thereafter. The same trial would be needed to look at the cost of treatment between the two methods. The MICN would need to show a significantly lower time to union and incidence of nonunion to justify the expense of the implant, a result which may vary between the tibia and femur. Such a study is the only way to truly know which patients require the MICN technique.

## Conclusion

The magnetic intramedullary compression nail has resulted in the union of several difficult to treat fracture nonunion cases. Confirming that enough compression has been applied can be inferred from the presence of locking bolt bending on radiographs. The ideal angle of screw bending needs to be further delineated and may be correlated to rate of consolidation, a finding that could not be proven from this small series. Proximal tibia metaphyseal nonunions may be ill-suited for this treatment due to inability to control deformity. Weight bearing as tolerated ambulation did not negatively impact the results and is our standard rehabilitation protocol. Compression can be administered in many patterns. We recommend establishing early compression and adding more as needed during follow-up. Staged lengthening with the same internal lengthening nail is a future option but may require slow distraction. The lessons learned from this experience will help us understand docking site compression when using a magnetic IM bone transport nail in the near future.
